# Assessment of the Exposure of Turkey Farmers to Antimicrobial Resistance Associated with Working Practices

**DOI:** 10.3390/vetsci6010013

**Published:** 2019-02-01

**Authors:** Giorgio Franceschini, Marta Bottino, Ilary Millet, Elisa Martello, Francesca Zaltron, Anna Rosa Favretto, Nicoletta Vonesch, Paola Tomao, Alessandro Mannelli

**Affiliations:** 1 Department of Veterinary Sciences, University of Turin, 10124 Turin, Italy; marta.bottino@unito.it (M.B.); ilary.millet@unito.it (I.M.); martello.elisa@gmail.com (E.M.); 2 Department of Law and Political, Economic and Social Sciences, University of Eastern Piedmont, 15121 Alessandria, Italy; francesca.zaltron@asie.it (F.Z.); favretto@asie.it (A.R.F.); 3 Department of Medicine, Epidemiology, Occupational Hygiene and the Environment, National Institute for Insurance against Accidents at Work (INAIL), 00078 Monte Porzio Catone (Rome), Italy; n.vonesch@inail.it (N.V.); p.tomao@inail.it (P.T.)

**Keywords:** antimicrobial resistance, animal farms, farmers, workers, risk assessment, FMEA

## Abstract

The objective of the present study was the identification of farming practices in the production of turkeys for human consumption, and their ranking in terms of the occupational probability of exposure to antimicrobial resistant (AMR) bacteria, for farm workers. We gathered evidence and data from scientific literature, on risk factors for AMR in farmers, and on the prevalence of those hazards across farming phases. We administered semi-structured interviews to public and private veterinarians in Northern Italy, to obtain detailed information on turkey farming phases, and on working practices. Data were then integrated into a semi-quantitative Failure Modes and Effect Analysis (FMEA). Those working practices, which are characterized by direct contact with numerous animals, and which are carried out frequently, with rare use of personal protection devices resulted as associated with the greatest probability of exposure to AMR. For methicillin resistant *Staphylococcus aureus* (MRSA), these included vaccination and administration of any individual therapy, and removal and milling of litter, given the exposure of farmers to high dust level. Indeed, levels of occupational exposure to MRSA are enhanced by its transmission routes, which include direct contact with animal, as well as airborne transmission. Level of exposure to extended spectrum beta lactamase (ESBL) is more strictly associated with direct contact and the oral-fecal route. Consequently, exposure to ESBL resulted and associated with the routinely tipping over of poults turned on their back, and with the individual administration of therapies.

## 1. Introduction

Microbiological risk assessment is specifically suited to provide scientific evidence to guide prevention. In antimicrobial risk assessment (ARRA), the association between antimicrobial use in animals, and the emergence of resistant organisms in humans can be evaluated [1]. Based upon the World Organization for Animal Health (OIE) framework, release assessment describes the processes underlying the occurrence of AMR in the food production chain, whereas exposure of humans can be associated with the consumption of food of animal origin [2]. Occupational exposure to AMR is also possible. In fact, animal farmers can be exposed to antimicrobial resistant (AMR) agents during working activities [3,4,5,6], and epidemiological studies showed that carriage of AMR bacteria in farm workers was associated with carriage in animals, and with contamination of the farm environment [3,7,8,9].

Among poultry farm workers, turkey farmers might be characterized by a particularly high probability of exposure to AMR. In fact, prevalence of resistance to antimicrobials in turkeys is of the same level of magnitude as in broiler production [10]. However, the production cycle is longer for turkeys than for broilers, and working practices are more complex and might involve more close contact with animals (for example, for the administration of therapies) and with the farming environment (i.e., litter milling and removal).

In this study, we applied a semi-quantitative ARRA to a portion of the risk pathway for farm workers. Specifically, we focused on the probability of exposure to AMR during farm working practices, by combining prevalence estimates of AMR in animals and in the farm environment (release assessment), with the probability of contacts of humans with AMR during each practice, which is part of the animal production process. The risk question was: Which working practices are associated with the greatest probability of exposure to AMR in turkey farmers? Our objective was to guide potential risk mitigation measures, including appropriate communication to farmers. Since we combined data on prevalence and on risk factors for AMR from different countries, with information on farming practices in Northern Italy, results of our study should be supported by more thorough investigations, including laboratory analysis, and the study of practices, in a well-defined reference population. Indeed, a major objective of this article was to present and apply, to AMR, the FMEA methodology, which was originally developed to identify possible failures in manufacturing processes, or in products or services. Subsequently, it has been extended to other areas, including food safety, such as the production of salmon, pork, dairy products, and milk pasteurization [11,12]. By using a modified FMEA method, we were able to deal with data on ordinal scales, which were then combined by using logical functions. Moreover, the same method can be adapted to different animal production systems, taking into account varying epidemiological and farming conditions. 

## 2. Materials and Methods 

### 2.1. Hazard Identification

Livestock-associated methicillin resistant *Staphylococcus aureus* (LA-MRSA), and extended spectrum beta lactamase (ESBL) were considered as the hazards of interest, mostly based upon their occurrence in the turkey production, as reported in scientific publications. *S. aureus* is widespread in humans and in animals, where it can cause infections of varying severity [13,14,15]. Methicillin resistance is mainly due to the acquisition of the mecA gene, encoding a β-lactam low affinity penicillin binding protein (PBP) called PBP2a [16]. Animals can act as a reservoir for LA-MRSA in people, and infection may occur via direct contact with animals, or indirect routes, such as, for example, dust in animal farming environment [17]. ESBL are a group of bacterial enzymes, which are able to inactivate beta-lactam antibiotics, namely, penicillin, and 2nd, 3rd, and 4th-generation cephalosporins [18]. Furthermore, ESBL might be associated with resistance to other antimicrobials, such as fluoroquinolones, aminoglycoside, and trimetroprim-sulfamethoxazole [19]. ESBL are produced by gram-negative bacteria, such as Enterobacteriaceae. CTX-M, SHV e TEM are ESBL, which are the most common in animals [16,20], and can be transferred among different bacteria. As a consequence, ESBL can be transferred from bacteria of animal origin to other bacteria, which are present in the human digestive tract, and which might cause infection in humans, although human strains caused by consumption of, e.g., cephalosporins to humans, are also circulating [21].

### 2.2. Exposure Assessment

We carried out two separate literature reviews, to collect information on: a) Risk factors for the transmission of AMR agents from animals and the farm environment, to farm workers, and b) prevalence of the selected hazards in animals and in the farming environment. Subsequently, we integrated these data with the reconstruction of phases of the production of turkeys for human consumption, and with the analysis of working practices, as obtained by previous publications and through semi-structured interviews to key informants. Finally, in a modified FMEA, we identified and assigned criteria to rank working practices, in terms of the probability of exposure of farm workers to AMR.

#### 2.2.1. Literature Review of Risk Factors for Farm Workers

A literature review on the association between AMR in animal farms, and in farm workers was carried out, with the following objectives: a) To obtain evidence of exposure of farm workers to AMR, during working practices; and b) to collect information on risk factors for exposure, as a contribution to the identification of evaluation criteria of working practices. Scientific articles, regarding risks factor in farming, MRSA, and ESBL were selected from a wider literature search, which was previously carried out on both poultry and swine production, and a range of AMR determinants, as part of an ongoing project (Martello et al. unpublished). Briefly, in this project relevant articles were retrieved from Pubmed and Scopus databases on the 27th of October and on the 6th of November, 2017, respectively. Papers published before the year 2000 were not included in the review. The following free string terms were used in Pubmed:

(Esbl OR MCR-1 OR carbapenemase OR (pAmpC) OR AmpC OR mrsa OR (bla AND CTX-M-1) OR (antimicrobial AND resistance) OR AMR) AND ((pig OR swine OR sow OR hog OR poultry OR fowl OR chicken OR hen OR turkey OR rooster OR avian OR farm) and (farmers OR workers)); and in Scopus: (Esbl OR MCR-1 OR carbapenemase OR AmpC OR mrsa OR (bla AND CTX-M-1) OR (antimicrobial AND resistance) OR AMR) AND (pig OR swine OR sow OR poultry OR fowl OR chicken OR turkey OR avian OR farm) and (farmers OR workers).

Given the specific objectives of our case study, we selected and used a subset of papers only based on risk factors, and on MRSA and ESBL, as antimicrobial determinants [22]. Association, between animals and the farming environment with the infection in farmers, was assessed based upon microbiological, or epidemiological evidence. Microbiological evidence consisted of the characterization of microbial agents, and subsequent confirmation of identity of microbial strains in farm sources, and in farmers [23]. Epidemiological evidence was based on association parameters, such as relative risks, odds ratios, or prevalence ratios. 

#### 2.2.2. Literature Review for AMR Prevalence in Turkey Farms 

To estimate the frequency of occurrence of AMR in animals and in the farming environment, prevalence of the selected AMR determinants (MRSA and ESBL) was estimated from data, which were obtained by a second literature review. Relevant articles were retrieved from Pubmed databases on 12 February 2018. Given recent advances in laboratory diagnosis in the specific field, papers published before the 2012 were not included in the review. Furthermore, only articles reporting data from Europe were considered. 

We used (MRSA OR ESBL) and turkey as a combination of string terms. Prevalence of AMR determinants was estimated by extracting from selected publications, the number of tested sampling units (animals or environmental substrates) and the number of positive units, for each data collection activity, and by the farming phase. Logistic regression analysis was used to estimate prevalence and 95% confidence interval (95% CI) of positive results to diagnostic tests, by the farming phase. When several data collection activities on the same agent and farming phase were recorded, non-independence of results within the same data collection activity was taken into account by using Generalised Estimating Equation (GEE, GENMOD procedure, SAS^®^), yielding inflated standard error and producing wide 95% CI. Subsequently, it was decided to subdivide pre-established prevalence ranges into four levels, so as to use this data in the final calculation of probability level of exposure for each work practice. The levels have been defined, as shown in Table 1. For their subdivision, it was decided to assign a prevalence range of 20% for each level, except for Level 4, where a longer interval has been adopted due to the fact that prevalences of more than 60% were considered as indicative of maximum evidence. 

#### 2.2.3. Information on Phases and Working Practices in Turkey Farming for Animal Consumption 

We obtained detailed descriptions of phases of the production of fattening turkeys, and of working practices, from a previous, online publication by the National Institute for Insurance against Accidents at Work (INAIL) [24]; it was also used as a template for the collection of further details on working practices in turkey farming in Northern Italy. For this aim, we administered semi-structured interviews [25] to the following key informants: Two public veterinarians, with official responsibility for poultry health, two private veterinary practitioners, working in the assistance to poultry production. Semi-structured interviews are flexible tools, which allowed the discussion of details and issues, which may arise in the course of the interview. In particular, we asked the following open-ended questions: a) "what are the main phases during an entire fattening turkey production cycle?” b) “What are the working practices associated with these main phases?”. 

The interviews were recorded, and subsequently analyzed using the thematic analysis method [26], to identify any detail regarding working practices in the different phases of fattening turkey production cycle. The interpretation and synthesis of the material from interviews, together with results of literature review of risk factors of AMR in farm workers, were integrated, and further discussed, to select criteria to be applied into a semi-quantitative FMEA, to rank working practices, in terms of the probability of exposure of farm workers to AMR. Once selected, those criteria were the object of a telephone interview to a farmer in Northern Italy, to obtain specific information on a real case-study for the subsequent FMEA computation.

#### 2.2.4. Failure Modes and Effect Analysis (FMEA)

FMEA is based upon a semi-quantitative evaluation of risks. In a traditional approach, the risk levels of the potential failures are identified by calculating a risk priority number (RPN), which is obtained by multiplying the frequency of occurrence of each failure (O), by the severity of consequences (S), and by the possibility of detecting and controlling the failure (D), before consequences take place [27]. Potential failures are ordered in terms of O, S, and D. In calculating RPN by the original FMEA method, the assigned ranks on the three indexes are interpreted as being numbers. Therefore, information, which is gathered on the qualitative scales, and subsequently, used to order failures, is arbitrarily interpreted on a quantitative scale. In other words, the original ordinal scale is transformed into a new, cardinal scale, which is characterized by a metric and by the integer number composition properties [28].

This arbitrary “promotion” of the scale properties brings about a series of problems in the RPN interpretation [29]. In more detail, the data numbering (scale levels) involves: a) The definition of the RPN on a formally wider scale than that of the three component indexes, which generates a fictitious increase of its resolution (the RPN dominium is from 1 to 1000); b) the assumption that the scales of the three S, O, and D indexes have the same metric and that the same danger level corresponds to the same values on different index scales; c) the assumption that the three failure mode indexes are all equally important; and d) the possibility of identifying, with the same RPN, situations characterized by different danger index levels. For example, the condition assigning to (S, O, and D) indexes the values (10, 1, 1) is considered at the same level as (5, 2, 1). Both situations determine an RPN = 10. The numeric data interpretation simplifies the RPN calculation, but it also increases the risk of moving its meaning away from the logic of the risk assessment team that supplied the figures. The numbering—acknowledging “metrological properties” higher than actually possessed by collected information—can therefore cause a “distortion” effect, which can partially or completely distort the contents.

We used a modified FMEA, and adapted the corresponding terminology, to the semi-quantitative assessment of the probability of exposure of workers to AMR. Moreover, we used logical functions to combine the components of FMEA, which we developed for our application. Specifically, the estimated prevalence (P) of AMR in the farm workplace (release assessment phase), in animals, or in the farm environment, was combined with the probability of exposure (E, exposure assessment phase) of farm workers to AMR, during each practice, to achieve a ranking of working practices, in terms of the probability of exposure of workers to AMR. 

FMEA indexes were interpreted as evaluation criteria *g_j_* (with *j* = 1,…, n), which were used to rank working practices, as potential risk modes, *a_i_* (with *i* = 1,…, m) in terms of priority level of exposure of workers to AMR. The evaluation criteria, *g_j_*, were selected by discussion among the authors, based upon the analysis of semi-structured interviews of key informants, and of literature review (see next section). 

The method considers each FMEA index as a “fuzzy” subset over the set of alternatives to be selected. The grade of membership of alternative $\left(g_{j}\left(a_{i}\right)\right)$ indicates the degree to which *a_i_* satisfies a generic evaluation criterion. The method suggests a two-step procedure [28].

(1) Aggregations of evaluations expressed on each criterion for a given alternative *a_i._*
  (1) $$RPC\left(a_{i}\right)=\underset{j}{\min}\left[Max\left\{Neg\left(I\left(g_{j}\right)\right),g_{j}\left(a_{i}\right)\right\}\right]$$
  where:
  $RPC\left(a_{i}\right)$ is the *Risk Priority Code* for the potential risk mode (or working practice) *a_i_*.
  $I\left(g_{j}\right)$ is the importance associated with each of the evaluation criteria *g_j_*.
  $Neg\left(I\left(g_{j}\right)\right)$ is the negation of the importance assigned to each of the criteria.
  The negation of an *s*-point ordinal scale is calculated as follows [30,31]:
  (2) $$Neg\left(L_{i}\right)=L_{s-i+1}$$
  where *L_i_* is the *i*th level of the scale.
(2) Determination of the work practice with the maximum risk priority code (a*)
  (3) $$RPC\left(a^{*}\right)=\underset{a^{i}\in A}{\max}\left\{RPC\left(a_{i}\right)\right\}$$
  where *A* is the set of potential modes of potential risk.

It is worth noting that, as appears in Equation (1), $RPC\left(a_{i}\right)$ is also defined on an ordinal scale with 10 levels of the same type as those used for evaluations of the indexes. The inspiring logic of the adopted method (Equation (1)) is that of giving more weight to the criteria that are considered as the most important by the analyst/decision maker [30,31].

If two or more work practices have the same *RPC* we may obtain a more detailed discrimination considering the supplementary indicator or tie-break index: $T\left(a_{i}\right)=DimA\left(a_{i}\right)$, being $DimA\left(a_{i}\right)$ the number of elements contained in the set $A\left(a_{i}\right)$, and $A\left(a_{i}\right)=\left\{g_{j}\left(a_{i}\right)|g_{j}\left(a_{i}\right)>RPC\left(a^{*}\right)\right\}$. This term represents a second-step investigation for establishing a measure of the dispersion of criteria, related to a specific working practice, around the *RPC* index. It provides an estimation of how many important criteria with high evaluations, compared with the calculated *RPC*, are present in the evaluation of each working practice.

We note that, in Equation (1), we are implicitly assuming a logic to satisfy all of the characteristics that are important. The term $Max\left\{Neg\left(I\left(g_{j}\right)\right),g_{j}\right\}$ indicates a value for a given criterion to the statement “if the criterion is important, then it has a high score”. Thus, we see that low-importance criteria have little effect on the overall “score”.

Equation (3) allows the selection of working practices with the maximum *RPC* value. The rationale of the procedure is to consider the most potentially dangerous working practices to be those with the highest evaluations on the most important criteria. When two or more practices have the same ranking, we provide a more meticulous selection with $T\left(a_{i}\right)$ index. $T\left(a_{i}\right)$ defines, for each working practice, the cardinality of the total number of “equivalent” risk levels associated with all criteria. While the traditional FMEA is not able to manage situations in which characteristic indexes have different importance, this approach allows to differentiate the relative importance of the severity, occurrence, and detection indexes.

## 3. Results and Discussion

Following a selection of papers from the literature review on risk factors for AMR in farm workers, five articles were selected regarding risk factors of MRSA for turkey farm workers. Fourteen articles were selected regarding risk factors for ESBL in workers (four on turkey farming), as shown in Table 2. The criteria for inclusion of the articles, although not systematically, were as follows: In Europe, from 2011 to 2018 and with reference to risk factors involved in the transmission of AMR to humans.

As a summary of main findings, prevalence of MRSA in persons working in turkey farms is much higher than the prevalence in the general population in Germany, and in the Netherlands [5,9]. Furthermore, people working in turkey farms, especially those working in the barn on a regular basis have an increased risk of being colonized with MRSA compared to the general public, family members and other farm workers. In the Netherlands, farmers’ occupational exposure was indicated by genome mapping of indistinguishable MRSA in animals and humans, in two turkey farms [5]. Physical contact with live turkeys and entering the poultry house was a relevant risk factor [5,9]. Given the scarcity of information on ESBL in turkey farmers, we used information from a study on broilers (*Gallus gallus*), where an increased risk of exposure to ESBL/AmpC-producing *E. coli* was found for people spending more than two hours per day in broiler houses [40]. Detailed information on working practices in turkey or poultry farms as risk factor for workers’ exposure to AMR, and on protective measures was, generally, very limited in scientific literature. While animal farm workers may acquire MRSA or ESBL from sources outside of the farm [5], there is strong evidence of the possibility of transmission of these agents from animals and the farming environment to workers. Based upon such a conclusion, we proceeded into the identification and semi-quantitative evaluation of farming practices, in terms of the probability of exposure of workers.

The literature search on prevalence of MRSA and ESBL in turkey farms yielded six articles for MRSA and four articles for ESBL in turkey farms. However, data on the number of samples tested and positive samples were only available in four articles for MRSA and no articles for ESBL. The latter did not subdivide the prevalence by the breeding phase and therefore were not used in the final study, as summarized in the Table 3.

All the publications used for the MRSA prevalence estimation levels, used microbiological investigation techniques for the mecA gene characterization in Staphylococcus aureus.

To conclude, the estimates of MRSA prevalence in animals and environmental substrates, respectively, are given in Table 4 and Table 5. Prevalence levels are included, based upon ranges of prevalence, as shown in Table 1. 

Prevalence of MRSA in turkeys increases with subsequent breeding phase, and with an increasing age of animals, from Level 2 in the first breeding phase, Level 3 in the intermediate phase to Level 4 in the last one. Prevalence of MRSA in environmental substrates passed from Level 2, in earlier phases, to Level 4, when turkeys are older than 100 days. 

Working practices, which we identified, based upon information from INAIL, and from the analysis of interviews to key informants, are listed, and briefly described in Table 6. 

Subsequently, by integrating information from scientific literature and semi-structured interview, the following FMEA evaluation criteria were identified: a) Type of contact with potential sources of the hazard (animals and farm environment), b) working hours, as a measure of duration of exposure; c) use of personal safety devices (PSD); and d) number of animals per operator, as a measure of intensity of exposure. Assigned levels of exposure to AMR for the above criteria are summarized in Table 7. 

It was decided to assign different levels of importance to each of the criteria on the basis of the potential transmission routes of each determinant (MRSA, ESBL). A 4-level scale of importance was used for each evaluation criteria. On the basis of scientific literature and expert opinions, importance of criteria, for each AMR (MRSA, and ESBL) is summarized in Table 8.

P.S.D are considered very important for MRSA as there are two possible transmission routes for these agents: Aerogen and direct contact [3,6]. The time spent inside the box, as emerged from the literature [6,32,36], seems to greatly amplify the probability of exposure. The type of contact certainly affects the final risk, but not as much as the two previous indicators [32]. 

As far as ESBL is concerned, it is important to highlight P.S.D as the most important in preventing the transmission to humans, which mainly occurs by an oral-fecal route. In the same way, the type of contact is equally important in the dynamics of the infection [40,52]. The working hours [4,38] and the number of animals per operator [38,39,47] are of lesser relevance for the transmission of the determinants to humans. We, however, considered the number of animals per operator as more important for ESBL than for MRSA. In fact, since we hypothesized this criterion to be more relevant for transmission by direct contact, whereas, for MRSA, which can be transmitte by the aerial route, other criteria are relatively more important [34,35].

Levels of occupational exposure to MRSA and ESBL are shown in Table 9 and in Table 10, respectively. These were obtained, for each working practice, by equation 1 and those evaluation criteria, which are listed in Table 7.

Realistic values for each evaluation criterion, which were obtained by the telephone interview to a farmer in Northern Italy, who manages a breeding of 40,000 turkeys divided into three sheds, were obtained on working hours, use of P.S.D, number of animals per operator, and at the same time, to verify the type of contact for each working practice described in Table 6. These levels are shown in Table 7. 

Backhand of the poults, vaccination and administration of any therapies individually resulted as those working practices, which are associated with the greatest level of occupational exposure to MRSA (L_4_). This might be justified by potential transmission routes of MRSA, which include direct contact with animal as well as airborne transmission. Backhand of the poults resulted as that working practice, which is associated with the greatest level of occupational exposure to ESBL, which is mostly transmitted by direct contact and oral-fecal route. While results, which we obtained based upon only one farm, are plausible and consistent with MRSA and ESBL transmission routes, different ranking of practices may result from the application of the same method to different farms, due to variable evaluation criteria values. The collection of information from a probabilistic sample of farms would provide representative values, still allowing ranking of practice at the individual farm level. 

### Calculation of the Probability of Exposure

We were able to calculate a final priority code (probability level of exposure) for each work practice in the corresponding pharming phases in which it is carried out, considering the possible sources of MRSA transmission (animals and environment) and associating them to the level of occupational exposure that was calculated before, as reported in Table 11. In this regard, it is worth remembering that the levels of occupational exposure calculated in Table 9 and Table 10 in relation to selected AMR determinants, have been used as new evaluation criteria.

In summary, using the FMEA methodology again, we have three new evaluation criteria (Occupational exposure, animals’ prevalence, and environmental prevalence). Knowing MRSA’s possible transmission routes, it was decided to give to the new evaluation criteria the same level of importance. For ESBL, since there is no airborne transmission, the highest importance could be given to the level of occupational exposure and animals prevalence (Lev.4) and less to the environmental prevalence (Lev.2). In this final phase of the study, we only considered the prevalence of MRSA due to the scarcity of prevalence data on ESBL in turkeys. Table 11 shows the priority codes (Probability level of exposure) for MRSA.

Working practices associated with the greatest probability level of exposure to MRSA are those combining high levels of occupational exposure (L_3_ or L_4_) with relatively high levels of prevalence of MRSA in the farming environment and in animals. Specifically, the administration of therapies involves direct contact with animals, whereas litter milling and removal might involve both direct and airborne exposure to MRSA. Furthermore, practices, which are carried out in the last phase of fattening turkeys farming (>100 days) are associated with the greatest probability of exposure. Accordingly, data from scientific literature show a constant increase in the prevalence of this AMR agent both in animals and in the farming environment substrates until it reaches its maximum level in the last part of the farming cycle. 

## 4. Conclusions

In this study, we applied a modified FMEA approach, which is simple, intuitive, and automatable; it is also quite flexible, as it can be applied to the prioritization of working practices, for the exposure to AMR agents, in other types of animals breeding, beyond the presented case study. In this application, we only included working practices, without considering factors associated with farm structure and management, which might affect transmission of AMR agents in animals and to people. Consequently, preventive measures, which can be recommended based upon the results of this application, are mostly limited to the use of P.S.D. In particular, workers in farming of turkeys for human consumption should wear P.S.D when engaging with practices involving direct contact with animals and lengthy exposure to animal feces, such as litter treatment. Furthermore, to avoid frequent litter milling, particular attention should be payed to maintaining a dry litter, by an efficient ventilation of the farming environment, and by avoiding excessive water dripping from drinking lines. 

Turkey farmers should undergo constant microbiological checks to monitor their actual level of infection with AMR. Further studies should be carried out to identify possible, alternative ways of carrying out certain working practices, to reduce contact with AMR determinants. Data collections and laboratory analysis of a representative sample of turkey farms in Italy would allow re-parametrizing the levels of the evaluation criteria, to obtain more realistic ranking of working practices and to compare different farms. The results could be used to devise farm-specific prevention measures. 

## Figures and Tables

**Table 1:** Subdivision of the prevalence’s of MRSA, or ESBL, in animals, or in the farming environment, in ranges on a scale of 4 levels. Prevalence’s greater than 60% were assigned maximum level.

Levels	Range of Prevalence
1	0–20%
2	20–40%
3	40–60%
4	>60%

**Table 2:** Main characteristics of included papers for risk factors of MRSA and ESBL for farm workers.

Paper	Reason for Paper Selection	Risk Factors	Country	Farming	Reference Population
[3]	Risk factors of MRSA	Working hours, P.S.D. (Personal Safety Devices)	The Netherlands	Pig	49 pig farms
[4]	Risk factors of ESBL	Type of contact with potential sources of ESBL, Working hours.	The Netherlands	Pig	40 pig farms
[5]	Risk factors of MRSA	Type of contact with potential sources of MRSA.	The Netherlands	Turkey, Duck	10 duck farms, 10 turkey farms
[6]	Risk factors of MRSA	Type of contact with potential sources of MRSA.	Belgium, Denmark, The Netherlands	Pig	4 pig farms
[7]	Risk factors of MRSA	Working hours, Type of contact with potential sources of MRSA, P.S.D.	Germany	Pig, Cattle, Poultry	17 pig farms, 11 cattle farms, 4 chicken farms, 2 turkey farms (at least 50 pigs or cattle per farm and 10,000 birds per farm)
[9]	Risk factors of MRSA	Working hours, P.S.D.	Germany	Turkey	20 turkey farms (from 3000 to 20,000 birds per farm)
[13]	Risk factors of MRSA	Number of animals per operator, P.S.D, Working hours, Type of contact with potential sources of MRSA.	Germany	Pig, Chicken, Cattle, Turkey, Horse, Dog, Cat, Sheep/Goat, Roe	Not specified
[14]	Risk factors of MRSA	Type of contact with potential sources of MRSA, Number of animals per operator.	European countries	Pig, Veal calf	Not specified
[17]	Risk factors of MRSA	Working hours, P.S.D.	Denmark	Pig	6 swine farms
[32]	Risk factors of MRSA	Working hours, P.S.D.	The Netherlands	Pig, veal calf	87 pig farms, 49 veal calf farms
[33]	Risk factors of MRSA	Number of animals per operator, P.S.D., Working hours, Type of contact with potential sources of MRSA.	Germany	Turkey, Broiler	5 fattening turkey farms (from 10,000 to 36,000 birds per farm), 2 broiler fattening farms (from 35,000 to 352,000 birds per farm)
[34]	Risk factors of MRSA	Number of animals per operator.	Spain	Pig	9 fattening pig farms, 11 farrow to finish pig farms (from 180 to 10,000 animals per farm)
[35]	Risk factors of MRSA	Working hours.	The Netherlands	Pig, veal Calf	102 veal calf farms, 50 pig farms
[36]	Risk factors of MRSA	Type of contact with potential sources of MRSA, P.S.D., Working hours.	The Netherlands	Pig	49 farrowing pig farms
[37]	Risk factors of MRSA	Number of animals per operator, P.S.D.	Germany	Turkey	2 broiler farms (13,200 birds) 5 turkey farms (25,450 birds)
[38]	Risk factors of ESBL	Number of animals per operator, Working hours.	The Netherlands	Pig	40 pig farms (2388 animals)
[39]	Risk factors of ESBL	Type of contact with potential sources of ESBL, Working hours.	Denmark	Pig	39 pig farms (20 with no third- or fourth-generation cephalosporin use and 19 with previous frequent use were included)
[40]	Risk factors of ESBL	Type of contact with potential sources of ESBL, Working hours, P.S.D.	The Netherlands	Broiler	50 broiler farms (from 14,400 to 200,000 birds per farm)
[41]	Risk factors of ESBL	Number of animals per operator, Type of contact with potential sources of ESBL.	The Netherlands	Broiler	26 broiler farms (>30,000 broilers per farm)
[42]	Risk factors of ESBL	Type of contact with potential sources of ESBL.	Germany, The Netherlands	Pig	35 pig farms (550 animals)
[43]	Risk factors of ESBL	Type of contact with potential sources of ESBL, Working hours	Germany	Broiler	7 broiler fattening farms (from 48,000 to 360,000 birds per farm)
[44]	Risk factors of ESBL	Type of contact with potential sources of ESBL, Number of animals per operator	Sweden	Broiler	Not specified
[45]	Risk factors of ESBL	P.S.D.	Czech Republic	Turkey	40 turkey farms
[46]	Risk factors of ESBL	Type of contact with potential sources of ESBL.	Finland	Cattle, Pig, Broiler, Turkey	55 broiler farms, 7 turkey farms, 66 pig farms, 197 cattle farms
[47]	Risk factors of ESBL	Number of animals per operator, P.S.D.	Germany	Pig	47 pig farms
[48]	Risk factors of ESBL	Type of contact with potential sources of ESBL, P.S.D.	Great Britain	Turkey, Broiler	Broiler not specified, 442 turkey farms
[49]	Risk factors of ESBL	Working hours, Type of contact with potential sources of ESBL, P.S.D.	Germany	Pig, Cattle, Poultry	17 pig farms, 11 cattle farms, 4 chicken farms, 2 turkey farms (at least 50 pigs or cattle per farm and 10,000 birds per farm
[50]	Risk factors of ESBL	Type of contact with potential sources of ESBL, P.S.D.	The Netherlands	Broiler	2 broiler farms (1 conventional with 98 birds and 1 organic with 51 birds)

**Table 3:** Main characteristics of included papers for MRSA and ESBL prevalence in turkey farm workers.

Paper	Reason for Paper Selection	Country	Farming	Reason for Exclusion from Prevalence Estimation
[5]	MRSA prevalence in birds	The Netherlands	Turkey	The prevalence concerns more than one sample taken from different farms and not divided in breeding phases
[7]	MRSA prevalence in birds	Germany	Turkey	
[9]	MRSA prevalence in birds and in farming environmental substrates	Germany	Turkey	
[33]	MRSA prevalence in birds and in farming environmental substrates	Germany	Turkey	
[37]	MRSA prevalence in birds	Germany	Turkey	The prevalence concerns more than one sample taken from different farms and not divided in breeding phases
[45]	ESBL prevalence in farming environmental substrates	Czech Republic	Turkey	The prevalence concerns more than one sample taken from different farms and not divided in breeding phases
[46]	ESBL prevalence in birds	Finland	Turkey	The prevalence is 0
[48]	ESBL prevalence in farming environmental substrates	Great Britain	Turkey	The prevalence concerns more than one sample taken from different farms and not divided in breeding phases
[49]	ESBL prevalence in birds	Germany	Turkey	The prevalence is 0
[51]	MRSA prevalence in farming environmental substrates	Germany	Turkey	

**Table 4:** Prevalence estimates of MRSA in turkeys, in different farming phases, as estimated by Generalized Estimating Equations on data from the literature review.

Breeding Phases	N° of Data Collections	N° of Tested Animals	N° of Positive Animals	Prevalence (%)	CI Min	CI Max	Resulting Level ^1^
Not specified ^2,3^	2	25	2	8.00	0.77	49.19	1
0–70 days ^3^	4	48	17	35.41 *	23.28	49.76	2
70–100 days ^3^	8	96	43	44.79	29.91	60.66	3
>100 days ^3,4^	10	688	422	60.06	49.79	69.51	4

MRSA status was confirmed by PCR for the *mecA* gene encoding for resistance to methicillin * Results from ordinary logistic regression. ^1^ Resulting level based on prevalence ranges reported in Table 1. ^2^ [7]. ^3^ [33]. ^4^ [9].

**Table 5:** Prevalence estimates of MRSA in farming environment substrates, in different farming phases, as estimated by Generalized Estimating Equations on data from the literature review.

Breeding Phases	N° of Data Collections	N° of Tested Environmental Substrates	N° of Positive Environmental Substrates	Prevalence (%)	CI Min	CI Max	Resulting Level ^1^
Not specified ^2^	1	112	22	19.64 *	13.29	28.03	1
0–70 days ^3^	4	20	5	25.00 *	10.80	47.83	2
70–100 days ^3^	8	40	10	25.00	13.73	41.10	2
>100 days ^3,4^	10	90	58	64.33	49.86	76.59	4

* Results from ordinary logistic regression. ^1^ Resulting level based on prevalence ranges reported in Table 1. ^2^ [51]. ^3^ [33]. ^4^ [9].

**Table 6:** List of the main working practices in turkey farming, as obtained through semi-structured interviews to key informants, in Northern Italy.

Working Practices	Short Description
Litter preparation	Practice characterized by 2 distinct work sub – phases: (1) introducing and laying the bedding material in the farming houses. (2) housing for the technical equipment needed to create the weaning areas already provided with water in the drinking troughs and feed in the feeding troughs.
Discharge of the poults	At this stage the turkey chicks arrive and are unloaded from transport vehicles within the circle weaning areas which are delimited by a net on the litter tray surface.
Backhand of the poults	The tipping over of poults turned on their back; it is a manual activity carried out especially during the first 3 days of birds’ life.
Removal of weaning areas	The removal of the nets delimiting the circle areas and therefore the release of the animals for the entire available litterfall space.
Vaccination	This type of intervention can take a few days and not less than 3-4 workers because it is carried out inside the boxes and mobile barriers are used to isolate and channel groups of animals, which will then be taken individually and vaccinated with syringes.
Animals’ inspection	In addition to animal inspection, it also covers the daily check of the correct functioning of the plant elements, with particular reference to the distribution systems of feeders and drinking troughs.
Administration of any therapies individually	Treatments are carried out manually by the operator and involve the restraining of the animal.
Lap of the dead	In mortality control, the operator must walk the entire surface of the pits on a daily basis, visually assess the condition of the animals, report any abnormalities in their physical condition and take dead animals away from the pits. Dead animals are introduced into a cold store normally located on the external yards of the breeding site.
Litter milling	This activity consists of tipping the litter tray over, so that the surface is always dry.
Loading of turkeys	The operators convey the animals to the central entrance using mobile barriers made of metal material. The operators then manually insert the turkeys into the crates, which are then inserted into the lorry for transport to the slaughterhouse.
Litter removal	The activity consists of collecting and moving away from the attic of the boxes, all the material making up the spent litter, composed of animal catabolites and wood shavings or rice chaff in a single biodegradable residual product. Harvesting is carried out by an operator who, by operating a bobcat, collects the faeces and conveys them outside, where they are then loaded onto a vehicle for delivery onto agricultural land.
Washing at low or medium pressure	Washing is carried out by insufflation of water at low or medium pressure or mist, both in the rooms and in the equipment.
Washing at high pressure	Washing is carried out by insufflation of water at high pressure or mist, both in the rooms and in the equipment.
Disinfection	After washing, surfaces and equipment are treated with disinfectant. Specifically, once an aqueous solution of known content has been obtained, it is directly injected into the surfaces to be treated.
Maintenance	It includes minor interventions of ordinary maintenance to equipment.

**Table 7:** Levels of exposure of farm workers to AMR determinants, in turkey farming, for four evaluation criteria.

Levels (1 = Lowest, 4 = Highest)	Evaluation Criteria			
	Type of Contact with Potential Sources of AMR	Working Hours per Operator	Personal Safety Devices	Number of Animals per Operator
1	Entry into the shed in absence of animals (eg: box preparation, disinfection…)	<2 h	Wearing mask, gloves and eye glasses.	<2500
2	Operations carried out remotely by animals (ex: washing, maintenance…)	2–4 h	Wearing 2 out of 3 devices.	2500–5000
3	Contact with dejections (ex: bedding removal…)	4–6 h	Wearing 1 out of 3 devices.	5000–7500
4	Direct contact (ex: discharge of the poults, discharge turkeys, vaccinations, weighs …)	>6 h	Wearing no device.	>7500

**Table 8:** Levels of importance to each of the criteria.

Criteria	Importance Level (MRSA)	Importance Level (ESBL)
Type of contact	3	4
Work hours	4	2
P.S.D	4	4
Number animals per operator	1	2

**Table 9:** Level of occupational exposure to MRSA in descending order for each of the working practices in the fattening turkey farming, as obtained by a modified FMEA. When two or more practices have the same ranking, they are ordered according to the tie-break rule.

Working Practices	Type of Contact (Level)	Working Hours per Operator (Level)	Personal Safety Devices (Level)	Number of Animals per Operator (Level)	Occupational Exposure (Level)
Backhand of the poults	4	4	4	4	4
Administration of any therapies individually	4	4	4	3	4
Vaccination	4	4	4	3	4
Litter removal	3	4	3	4	3
Removal of weaning areas	3	3	4	4	3
Discharge of the poults	3	3	4	4	3
Litter milling	3	3	3	4	3
Lap of the dead	4	2	3	4	2
Litter preparation	1	4	4	1	2
Charge of the turkeys	4	2	3	2	2
Maintenance	2	2	3	4	2
Washing at high pressure	3	4	2	1	2
Washing at low or medium pressure	2	4	2	1	2
Animals’inspection	2	1	4	4	1
Disinfection	1	2	1	1	1

**Table 10:** Level of occupational exposure to ESBL in descending order for each of the working practices in the fattening turkey farming, as obtained by a modified FMEA. When two or more practices have the same ranking, they are ordered according to the tie-break rule.

Working Practices	Type of Contact (Level)	Working Hours per Operator (Level)	Personal Safety Devices (Level)	Number of Animals per Operator (Level)	Occupational Exposure (Level)
Backhand of the poults	4	4	4	4	4
Vaccination	4	4	4	3	3
Administration of any therapies individually	4	4	4	3	3
Litter removal	3	4	3	4	3
Removal of weaning areas	3	3	4	4	3
Discharge of the poults	3	3	4	4	3
Lap of the dead	4	2	3	4	3
Litter milling	3	3	3	4	3
Charge of the turkeys	4	2	3	2	3
Animals’inspection	2	1	4	4	2
Maintenance	2	2	3	4	2
Washing at high pressure	3	4	2	1	2
Washing at low or medium pressure	2	4	2	1	2
Litter preparation	1	4	4	1	1
Disinfection	1	2	1	1	1

**Table 11:** Probability level of exposure to MRSA in descending order for each of the working practices in the fattening turkey farming, as obtained through a modified FMEA. When two or more practices have the same ranking they are ordered according to the tie-break rule.

Working Practices	Level of Occupational Exposure (from Table 5)	Level of Animals Prevalence	Level of Environmental Prevalence	Level of Probability of Exposure
Administration of any therapies individually (>100 days)	4	4	4	4
Litter milling (>100 days)	3	4	4	3
Litter removal (>100 days)	3	4	4	3
Lap of the dead (>100 days)	2	4	4	2
Charge of the turkeys (>100 days)	2	4	4	2
Washing at high pressure	2	4	4	2
Washing at low or medium pressure (>100 days)	2	4	4	2
Maintenance (>100 days)	2	4	4	2
Administration of any therapies individually (70–100 days)	4	3	2	2
Administration of any therapies individually (0–70 days)	4	2	2	2
Vaccination (0–70 days)	4	2	2	2
Backhand of the poults (0–70 days)	4	2	2	2
Litter milling (70–100 days)	3	3	2	2
Discharge of the poults (0–70 days)	3	2	2	2
Removal of weaning areas (0–70 days)	3	2	2	2
Lap of the dead (70–100 days)	2	3	2	2
Litter milling (0–70 days)	3	2	2	2
Maintenance (70–100 days)	2	3	2	2
Maintenance (0–70 days)	2	2	2	2
Lap of the dead (0–70 days)	2	2	2	2
Litter preparation (0–70 days)	2	2	2	2
Disinfection (>100 days)	1	4	4	1
Animals’inspection (>100 days)	1	4	4	1
Animals’inspection (70–100 days)	1	3	2	1
Animals’inspection (0–70 days)	1	2	2	1
